# Treatment of chronic invasive fungal sinusitis with voriconazole in an HIV patient

**DOI:** 10.1186/1758-2652-13-S4-P190

**Published:** 2010-11-08

**Authors:** A Ho, G McGarry, E Peters

**Affiliations:** 1 The Infection, Tropical Medicine and Counselling Service, Brownlee Centre, Gartnavel General Hospital, Glasgow, UK; 2 Glasgow Royal Infirmary, Department of Ear, Nose and Throat, Glasgow, UK

## Purpose of the study

Chronic invasive fungal sinusitis is a rare condition, and the conventional treatment surgical debridement and systemic antifungal therapy such as amphotericin B. Recently, voriconazole has demonstrated superior efficacy in the treatment of invasive aspergillosis, compared to amphotericin B. However, its use in invasive fungal sinusitis in an HIV patient has not been reported.

## Methods

We describe a case of chronic invasive fungal sinusitis in a patient with HIV infection successfully treated with surgical clearance and a prolonged course of oral voriconazole.

## Summary of results

A 39 year old Malawian lady presented to the Ear, Nose and Throat (ENT) surgeons with recurrent nasal polyps and was noted to have proptosis in April 2009. She had had a previous polypectomy in 2007, which grew *Streptococcal pyogenes* and *Aspergillus flavus*. MRI sinuses in May 2009 demonstrated extensive soft tissue mass involving right maxillary, bilateral ethmoid and frontal sinuses, causing right axial proptosis. Dural enhancement suggested intracranial extension, and there was erosion into pituitary fossa posteriorly. Her only complaint was epiphora, with no visual disturbance. An HIV test was positive. Baseline CD4 count was 395 cells/cm^3^, and viral load 103,469 copies/ml. She underwent endoscopic sinus surgery to debulk tissue from her sinuses. Grocott staining of biopsy specimen demonstrated presence of fungal hyphae, and tissue culture grew *Aspergillus flavus*. She was commenced on oral voriconazole 300mg b.d. Due to potential drug interactions with non-nucleoside reverse transcriptase inhibitors (NNRTI) and protease inhibitors (PI), she was treated with tenofovir, emtricitabine and raltegravir. Follow-up MRI after 4 months of therapy showed marked improvement with resolution of proptosis. She has been on voriconazole for 12 months with no progression.

## Conclusions

Invasive fungal sinusitis has been rarely reported in HIV patients, but recent case reports in non-HIV immunocompromised patients have shown a favourable response to new triazoles. It has previously been associated with high mortality even with amphotericin therapy. There are important potential interactions with antiretroviral (ARV) drugs. The optimal duration of treatment for immunocompromised patients is unclear.

